# Nuclear Distribution of the Chromatin-Remodeling Protein ATRX in Mouse Early Embryos during Normal Development and Developmental Arrest In Vitro

**DOI:** 10.3390/life14010005

**Published:** 2023-12-19

**Authors:** Irina O. Bogolyubova, Zhuldyz K. Sailau, Dmitry S. Bogolyubov

**Affiliations:** 1 Institute of Cytology of the Russian Academy of Sciences, St. Petersburg 194064, Russia; ibogol@mail.ru; 2 PERSONA International Clinical Center for Reproductology, Almaty 050060, Kazakhstan; zhuldyz.ks@mail.ru

**Keywords:** mouse embryos, ATRX, pericentromeric heterochromatin, major satellite DNA, two-cell block in vitro

## Abstract

The chromatin-remodeling protein ATRX, which is currently recognized as one of the key genome caretakers, plays an important role in oogenesis and early embryogenesis in mammals. ATRX distribution in the nuclei of mouse embryos developing in vivo and in vitro, including when the embryos are arrested at the two-cell stage—the so-called two-cell block in vitro—was studied using immunofluorescent labeling and FISH. In normally developing two- and four-cell embryos, ATRX was found to be closely colocalized with pericentromeric DNA sequences detected with a probe to the mouse major satellite DNA. The association of ATRX with pericentromeric heterochromatin is mediated by nuclear actin and reduced after the treatment of embryos with latrunculin B. When culturing embryos in vitro, the distribution pattern of ATRX changes, leading to a decrease in the association of this protein with major satellite DNA especially under the two-cell block in vitro. Taken together, our data suggest that the intranuclear distribution of ATRX reflects the viability of mouse embryos and their probability of successful preimplantation development.

## 1. Introduction

The early stages of mammalian development are accompanied by pronounced chromatin rearrangements in the embryo. A characteristic feature of zygotic pronuclei and embryo nuclei at the initial stages of cleavage is the condensation of heterochromatin around the so-called nucleolus precursor bodies (NPBs) [1], also called, in recent years, atypical nucleoli [2,3]. As a result, specific NPB-associated regions of heterochromatin are formed.

The emergence of the unique heterochromatin landscape in early mammalian embryogenesis involve all key epigenetic regulators that ensure DNA methylation, post-translational modifications of histones, the emergence of alternative histone forms and ATP-dependent chromatin remodeling [4]. The contribution of the latter process to specific heterochromatin rearrangements in early embryos has been studied in less detail compared to other epigenetic mechanisms. However, even rare reports convincingly demonstrate the functional importance of ATP-dependent chromatin remodeling for the development of a new organism [5].

Particular attention in this context is drawn to the chromatin-remodeling protein ATRX belonging to the SWI/SNF2 family, which received its name after describing its association with the ATR-X syndrome [6]. For the combination of ATPase and helicase activities, ATRX plays a critical role in a wide range of biological processes, including transcriptional regulation, DNA repair, and chromosome segregation [7].

The ATRX molecule contains several functionally significant domains [8], which ensure its interactions with chromatin and with other chromatin-associated proteins. A cysteine-rich domain containing a GATA-like domain and a plant homeodomain (PHD) in the structure—ADD (ATRX-DNMT3-DNMT3L)—is responsible for binding ATRX to H3. At this, the interaction of ADD with H3 is stimulated by the methylation of the latter on lysine at position 9 (H3K9me3), but is suppressed by the methylation of lysine at position 4 (H3K4me3). Further, ATRX contains an HP1α-interacting domain that mediates the interaction of ATRX with chromatin via the heterochromatin protein HP1α. Another region of the ATRX molecule—the Daxx interacting domain—underlies the interaction with the Daxx protein, another important functional partner of ATRX, which plays, in particular, an essential role in mammalian early development [9]. In addition, the ATRX molecule contains a domain that provides an interaction with N-methyltransferase EZH2 (enhancer of zeste homolog 2). EZH2 catalyzes the addition of methyl groups to the H3 lysine at position 27 [8]. Finally, an acidic patch of the ATRX molecule is required for the binding of H4 in the nucleosome to the surfaces of neighboring nucleosomes, which leads to compaction of the chromatin fibril [10].

ATRX is currently considered a central “caretaker” of the genome [11]. Its functions have traditionally been associated with maintaining the structure of repressed chromatin, including pericentromeric and telomeric sequences [12,13]. However, there is much experimental evidence suggesting that ATRX can also bind active regulatory elements in euchromatin, serving as a general facilitator of cell-specific transcriptional and epigenetic programs in both hetero- and euchromatin [14].

Available experimental data prove the importance of ATRX for the formation of a new organism. In particular, ATRX is required for the maintenance of chromosome stability during meiosis, since RNAi-derived ATRX-deficient mouse oocytes show abnormal chromosome morphology at the MII stage, as well as defects in chromosome segregation leading to aneuploidy [15,16]. In embryos, known epigenetic asymmetry of pericentric heterochromatin before the onset of the first cleavage division is largely conferred by an ATRX-dependent silencing of major satellite transcripts. Loss of ATRX leads to numerous double strand DNA breaks, significantly increasing the frequency of the centromeric recombination during mitosis [12].

ATRX is transmitted from an oocyte to zygote with maternal chromosomes [15] and subsequently exhibits dynamic changes of its intranuclear distribution during cleavage. In mouse zygotes, ATRX is localized to heterochromatic regions of the maternal but not paternal pronucleus immediately after fertilization [12]. By the end of the first cell cycle, ATRX is detected at the NPB periphery in both maternal and paternal pronuclei [17]. In addition to this pattern, large ATRX-containing heterochromatin aggregates are detected at the two-cell stage [18], which can be explained by the dynamics of the prochromocenter and chromocenter formation during mouse embryogenesis [19]. However, the predominantly diffuse distribution of ATRX described for the morula stage [18] cannot be explained by the dynamics of chromocenters. The characteristic ATRX redistribution in the nuclei of preimplantation mouse embryos also cannot be explained by changes in the transcriptional activity [18].

In this regard, the aim of this work was to study the intranuclear distribution pattern of ATRX and its colocalization with pericentromeric DNA sequences in preimplantation mouse embryos developing under different conditions. Considering the importance of the formation of the NPB-associated heterochromatin compartment containing pericentromeric and centromeric DNA for normal development [20,21], as well as the importance of ATRX for the structural maintenance of pericentromeric heterochromatin [11], one can assume that changes in the topological association of ATRX with pericentromeric heterochromatin may reflect disturbances in early development. To test this hypothesis, we analyzed the distribution of ATRX in the nuclei of embryos whose cleavage was arrested at the late two-cell stage—an in vitro two-cell block model.

The phenomenon of the two-cell block in vitro was described at the end of the 20th century, when in vitro fertilization technologies were rapidly being developed. It was found that human embryos—as well as the embryos of inbred animal lines—are not capable of full preimplantation development in standard media and stop their cleavage at the species-specific stage [22]. In mice, this developmental arrest occurs at the late two-cell stage. It is important to emphasize that in vitro blocked embryos retain their viability and do not show signs of degeneration within 3 days [23]. Moreover, it has been believed that a partial implementation of the development program continues in such embryos [23,24].

Here, we found that the distribution of ATRX is significantly different in mouse embryos in the two-cell block in vitro and in normally developing embryos. The close association of ATRX with pericentromeric heterochromatin, characteristic of two–four-cell embryos, is disrupted when embryos develop under suboptimal conditions in vitro. This suggests the ATRX distribution pattern can serve as a potential marker of embryo viability.

## 2. Materials and Methods

### 2.1. Embryo Collection

BALB/c mouse females obtained from the “Rappolovo” nursery (Leningrad Region, Russia) were used. Mice were administered with serum gonadotropin (Folligon, Intervet, Boxmeer, The Netherlands), 7.5 UI per animal, to stimulate ovulation. After 44–48 h, chorionic gonadotropin (hCG) (Chorulon, Intervet, Boxmeer, The Netherlands) was injected in the same dose and females were placed with fertile males; the mating success was assessed by the presence of vaginal plugs. The age of the embryos was counted from the hCG injection. Oviducts were removed into F-12 Ham medium containing HEPES (25 mM, pH 7.2–7.4) (Sigma-Aldrich, Stainheim, Germany). Embryos were isolated and collected in the same medium. If necessary, the embryos were treated with DNase (0.5 IU/μL; Sigma) or latrunculin B (1 μg/mL, Sigma) for 1 h at 37 °C.

To obtain embryos in the two-cell block in vitro, they were explanted 24 h after hCG. Embryos were cultured in a CO_2_ incubator under standard conditions (37 °C, 5% CO_2_) in an F-10 HAM medium with sodium bicarbonate (Sigma-Aldrich, Stainheim, Germany); control embryos were cultured in Continuous Single Culture Medium (CSCM, Irvin Scientific, Santa Ana, CA, USA). The general scheme of the experiments is shown in Supplementary Figure S1.

The animal work adheres to all ethical rules (3R principles). During the study, the animals were housed in the premises of the vivarium of the Institute of Cytology RAS, which was furnished with all the necessary equipment. Animals were sacrificed by a transcervical displacement of the cervical vertebrae, which complies with international principles for the humane treatment of laboratory animals.

### 2.2. Immunofluorescence Microscopy

Embryos were fixed for 40–60 min at room temperature in a 4% formaldehyde solution in PBS, which was prepared from paraformaldehyde. Then, the embryos were placed in 2% formaldehyde in PBS for at least 12 h at 4 °C. After rinsing in PBS, the embryos were placed in 0.5% Triton X100 (Sigma-Aldrich, St. Louis, MO, USA) in PBS for 10 min at room temperature to permeabilize the plasma membrane. After rinsing in PBS, the embryos were incubated in 10% fetal serum (Gibco Laboratories, Grand Island, NY, USA) for 10 min at room temperature to prevent non-specific antibody binding. Primary antibodies were mouse monoclonal (ab184228, Abcam, Cambridge, UK) and rabbit polyclonal (H-300, Santa Cruz Biotechnology) anti-ATRX antibodies, rabbit polyclonal anti-Daxx antibodies (M-112, Santa Cruz Biotechnology, Dallas, TX, USA), an anti-H3 (trimethyl K9) rabbit polyclonal antibody (ab8898, Abcam, Cambridge, UK), and a polyclonal antibody against the C-terminus of actin (A2066, Sigma-Aldrich, St. Louis, MO, USA), all diluted 1:100. Embryos were incubated with primary antibodies in a moist chamber at 4 °C for at least 12 h. After rinsing in PBS, embryos were placed in a solution of secondary antibodies for 90 min at room temperature. Primary antibodies were goat or donkey anti-rabbit or goat anti-mouse IgGs conjugated with FITC, Alexa-633, or Alexa-647, diluted 1:200. The embryos were embedded in Vectashield^®^ (Vector Laboratories, Burlingame, CA, USA) containing 0.5 μg/mL DAPI (Serva, Heidelberg, Germany) to stain the chromatin. Preparations were analyzed in a Leica TCS SP5 laser scanning confocal microscope equipped with a set of appropriate lasers and 40×/1.25 objective. To prevent the possible overlapping of the fluorescence spectra of dyes when several fluorochromes were used simultaneously, a sequential scanning was performed for each channel. For a qualitative analysis of the ATRX distribution pattern, at least 20 embryos per stage were analyzed. The images were merged using ImageJ 1.48v software, which also performed a colocalization analysis and measurement of Mean Gray Value. The correlation coefficient for dual channel images was calculated using the JACoP plugin. The statistical significance was assessed using the Mann–Whitney test.

### 2.3. Immuno-FISH

Embryos were fixed in 4% formaldehyde for no longer for than 30 min, after which they were washed in PBS and stained with anti-ATRX antibody alone or with a mixture of anti-ATRX and anti-DAXX antibodies, as described above (Section 2.2). After incubation in secondary antibodies and washing in PBS, the embryos were postfixed in a methanol–acetic acid mixture (3:1) on ice, then hydrated in a descending series of ethanol, and placed in a cold 2× sodium saline citrate (SSC). Denaturation was carried out in 70% formamide on 2× SSC, pH = 7.0, at 72 °C for 5 min. Next, the embryos were incubated in a hybridization probe solution in a hybridization buffer (50% formamide; 20% dextran sulfate; salmon sperm DNA 10 mg/mL; 2× SSC) for at least 12 h at 37 °C in a moist chamber. The TAMRA-labeled oligonucleotide probes to MaSat (5′-AGGACCTGGAATATGGCGAGAAA-3′) and MiSat (5′-TGTATATCAATGAGTTACAATGA-3′) were used for DNA–DNA FISH [25,26]. The embryos were then washed in 2× SSC for 10 min at 42 °C, in 1× SSC for 10 min at room temperature, and in 0.5× SSC for 5 min at room temperature; then, they were embedded in Vectashield^®^ with DAPI.

### 2.4. FRET

The samples were examined with an Olympus FV3000 confocal microscope equipped with 561- and 640-nm lasers. FRET AB procedure (Acceptor Photobleaching) was used. Areas of the samples not subjected to photobleaching were used as a negative control. Fluorochrome TAMRA was used as a donor, with Alexa 647 (647 donkey anti-rabbit IgG, Molecular Probes) as an acceptor. The FRET efficiency was calculated using the formula: E = (D_post_ − D_pre_)/D_post_; where D_post_—donor fluorescence intensity after acceptor photobleaching, D_pre_—donor fluorescence intensity before acceptor photobleaching.

## 3. Results

**Figure 1:**
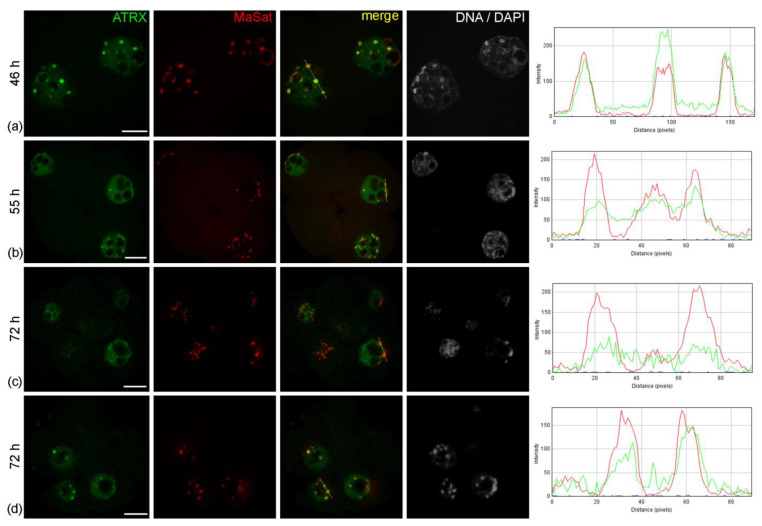
Dynamics of intranuclear distribution of ATRX in mouse embryos developing in vivo. (**a**) Late two-cell stage—46 h post-hGG; (**b**) four-cell stage—55 h post-hGG; (**c**,**d**) morulae—72 h post-hGG. Note the close association of ATRX with the major satellite DNA (MaSat) in the pericentromeric heterochromatin at the two–four-cell stage and decrease in ATRX–MaSat colocalization at the morula stage. RGB profile plots for the yellow lines in merged images are presented in the right column; green, ATRX; red, MaSat. Scale bars represent 10 μm.

**Figure 2:**
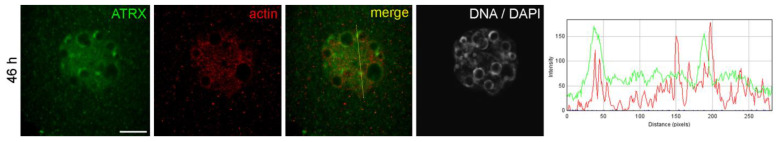
Colocalization of ATRX and actin in the nuclei of two-cell mouse embryos developing in vivo, 46 h post-hGG. RGB profile plot for the yellow line in the merged image is shown on the right; green, ATRX; red, actin. Scale bar represents 10 μm.

**Figure A1:**
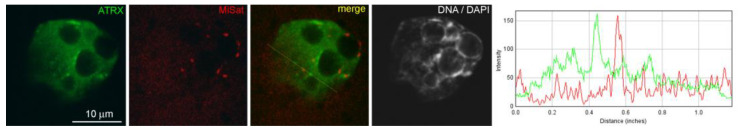
The nucleus of a two-cell mouse embryo (46 h post-hGG; in vivo development) after immuno-FISH for ATRX and minor satellite DNA (MiSat). RGB profile plot for the yellow line in the merged image is presented on the right; green, ATRX; red, MiSat.

**Figure A2:**
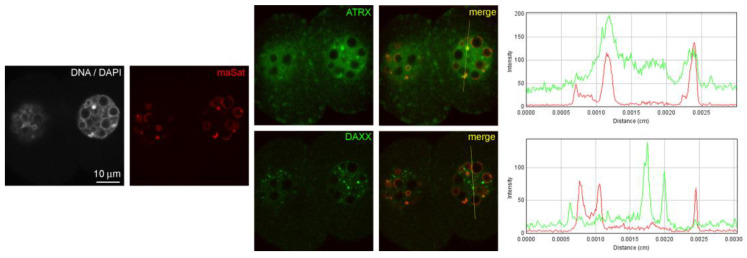
Simultaneous detection of ATRX, DAXX, and the major satellite DNA (MaSat) in mouse two-cell embryo developing in vivo (46 h post-hGG). RGB profile plots for the yellow lines in merged images are presented in the right column; green, ATRX (up) and DAXX (bottom); red, MaSat.

The nuclei of intact two-cell embryos developing in vivo—46 h after hGG—had clear zones of ATRX concentration associated with rims of heterochromatin around atypical nucleoli (NPBs) and with individual round clumps of heterochromatin (Figure 1a). The ATRX-containing heterochromatin regions also contained actin in well-detectable amounts (Figure 2). These ATRX-enriched zones were strongly colocalized with the regions of pericentromeric heterochromatin detected by DNA–DNA FISH (Figure 1a). The colocalization of ATRX with centromeric zones enriched in MiSat (Figure A1 in Appendix A), as well as the colocalization of the Daxx protein—the main functional partner of ATRX—with MaSat (Figure A2 in Appendix A), were much less pronounced, which proves the specificity of the association of ATRX with zones of pericentromeric heterochromatin.

A similar pattern of ATRX localization was also detected in four-cell embryos—55 h after hGG (Figure 1b). A different picture was typical for the nuclei of the morula—72 h after hGG. In this case, a diffuse distribution of ATRX was predominant throughout the nucleus, while the enrichment of MaSat-positive zones with ATRX became less prominent (Figure 1c). The results of the quantitative analysis of ATRX and MaSat colocalization are presented in Table 1. At the same time, the close association of ATRX and MaSat DNA remained in a small number (about 12%) of morulae (Figure 1d).

**Table 1:** Quantitative analysis of ATRX and MaSat colocalization in mouse embryos developing under different conditions.

Experimental Conditions	n	Period Post-hGG, Hours	Number of Blastomeres	Correlation Coefficient for Dual Channel Images	Representative Images
Normal development in vivo	10	46	2	0.490 ± 0.12	Figure 1a
	8	72	8–16	0.308 ± 0.044 **	Figure 1c,d
Normal development in vitro	4	48	2	0.225 ± 0.051 **	Figure 3b,c
	5	72	8–16	0.407 ± 0.131 **	Figure 4c
Two-cell block in vitro	4	48	2	0.249 ± 0.061 *	Figure 3a
	6	72	2	0.314 ± 0.062 *	Figure 4a,b

Significant differences from control two-cell embryos (46 h in vivo) are shown: * *p* ≤ 0.05, ** *p* ≤ 0.01.

**Figure 3:**
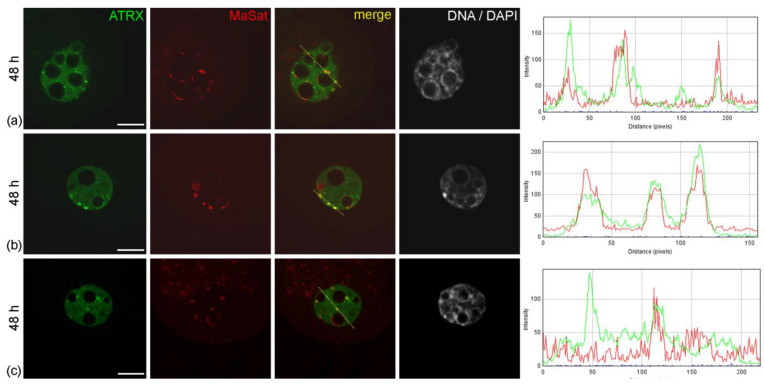
Intranuclear distribution of ATRX in mouse embryos developing in vitro for 24 h—which corresponds to 48 h post-hGG of normal development—in F-10 HAM medium (**a**) and Continuous Single Culture Medium (**b**,**c**). RGB profile plots for the yellow lines in merged images are presented in the right column; green, ATRX; red, MaSat. Scale bars represent 10 μm.

**Figure 4:**
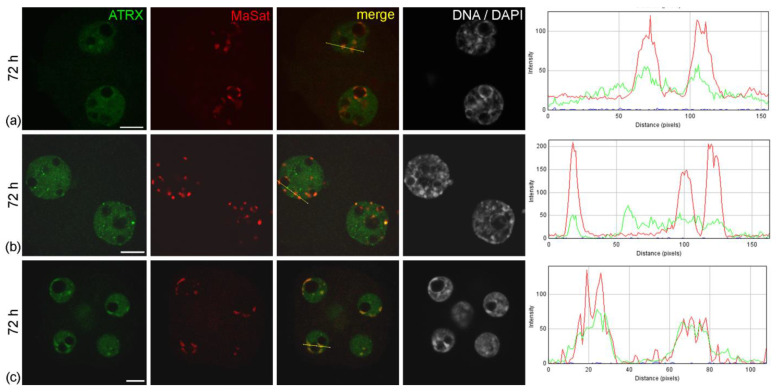
Intranuclear distribution of ATRX in mouse embryos developing in vitro for 48 h—which corresponds to 72 h post-hGG of normal development—in F-10 HAM medium (**a**,**b**) and Continuous Single Culture Medium (**c**). RGB profile plots for the yellow lines in merged images are presented in the right column; green, ATRX; red, MaSat. Scale bars represent 10 μm.

**Figure 5:**
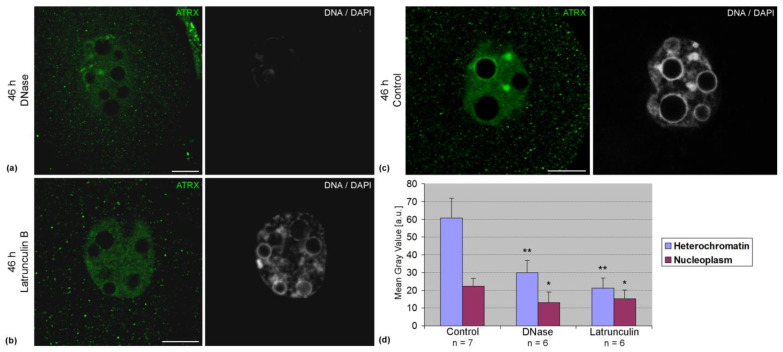
Changes in the intranuclear distribution pattern of ATRX in two-cell mouse embryos developing in vivo, 46 h post-hGG, after DNase (**a**) and latrunculin B treatment (**b**). (**c**) Control embryo. (**d**) The results of photometric image analysis. Values are the mean ± SD. * *p* < 0.05, ** *p* < 0.01. Scale bars represent 10 μm.

Association of ATRX with pericentromeric DNA sequences was confirmed by FRET analysis. In two-cell embryos, the average intensity of FRET in these zones was 8.4 ± 5.0% (n = 14). The treatment of embryos with DNase lead to a significant decrease in the intensity of ATRX staining (Figure 5a). Along with this, a decrease in the intensity of ATRX staining was also observed after the treatment of embryos with latrunculin B (Figure 5b). Photometric analysis confirmed the results of visual observations (Figure 5c).

When embryos were cultured in vitro, the pattern of ATRX distribution in their nuclei changed significantly. As early as 24 h after the start of cultivation—48 h after hGG—separate small ATRX-containing zones not associated with pericentromeric heterochromatin appeared in the nuclei of embryos developing in the F-10 HAM medium, instead of the typical distribution pattern described above (Figure 3a). Along with this, ATRX-less MaSat-positive zones were still detectable at the periphery of NPBs. Most control embryos developing in CSCM maintained the typical ATRX distribution pattern described above (Figure 3b). Discrete zones of ATRX localization not associated with the FISH signal, as well as the regions of MaSat DNA lacking ATRX, were detected only in individual control embryos (Figure 3c). However, the number of such embryos did not exceed 20%.

**Figure 6:**
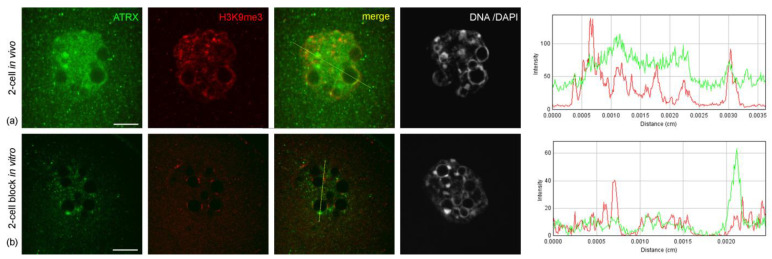
Immunodetection of ATRX and H3K9me3 in the nuclei of control (**a**) and blocked (**b**) two-cell embryos. RGB profile plots for the yellow lines in merged images are presented in the right column; green, ATRX; red, H3K9me3. Scale bars represent 10 μm.

The abnormal distribution pattern of ATRX in embryos arrested at two-cell stage persisted and intensified after 48 h of culture—i.e., 72 h post-hGG (Figure 4a,b). In addition, embryos in the two-cell block in vitro showed a significant change in the distribution pattern of H3K9me3—a major epigenetic mark of repressed chromatin—compared to control two-cell embryos developing in vivo. The intensity of the immunofluorescent signal for H3K9me3 in blocked embryos was noticeably lower and persisted only at the periphery of NPBs (Figure 6).

Embryos developing in vitro in CSCM can reach the morula stage after 48 h of culture. However, they demonstrated a different pattern of ATRX distribution compared to embryos of the same biological age developing in vivo. During in vitro development even in an enriched culture medium, ATRX accumulation in areas of pericentromeric heterochromatin was more pronounced, being observed more often than in morula nuclei during development in vivo (Figure 4c).

## 4. Discussion

Considering the importance of changes in the heterochromatin landscape for early mammalian development and the involvement of the chromatin remodeling protein ATRX in maintaining heterochromatin structure [7,12], we performed here an immunocytochemical analysis of the intranuclear distribution of ATRX in mouse embryos aged 46 to 72 h after hGG. The most prominent zones enriched in ATRX and closely colocalized with AT-rich sequences of MaSat DNA were found in embryos at the age of 46–55 h—i.e., during the second–third cell cycle. The second cell cycle plays a critical role in early mouse development, since at the end of the two-cell cleavage stage, the main events of zygotic genome activation (ZGA) are completed [27] and the processes of nucleolus reactivation begin [28,29]. In addition, pericentromeric heterochromatin is reorganized at this period, leading to the formation of chromocenters typical of a somatic cell [26]. Chromocenters are not detected in the pronuclei of the zygote and nuclei of embryos at the beginning of the second cell cycle, when pericentromeric sequences are organized around the NPBs and are parts of the heterochromatic rim [19].

The important role that ATRX plays in maintaining the structure of constitutive heterochromatin—including pericentromeric and telomeric sequences—is well documented [30]. Given that the artificial suppression of transcription does not lead to a pronounced change in the pattern of ATRX localization [18], it can be assumed that the dynamics of ATRX localization in two–four-cell mouse embryos are associated primarily with the reorganization of pericentromeric heterochromatin, and not with a change in the global level of transcriptional activity. At the same time, it cannot be excluded that ATRX during this period of development may be important for the regulation of the transcription of pericentromeric sequences. It is known that the formation of chromocenters at the two-cell stage of mouse development is accompanied by a burst of pericentric transcription [31], and only reverse pericentric transcripts are required for both heterochromatin reorganization and development beyond the two-cell stage [32]. In turn, ATRX—at least during the first cell cycle—is required for the silencing of major satellite transcripts in the maternal genome [12].

A decrease in the intensity of the fluorescent staining of ATRX-positive zones after the treatment of embryos with DNase confirms the close association of this protein with DNA. Indeed, the literature indicates the possibility of direct binding of ATRX to DNA, which is probably mediated by the basic alpha-helical region of the GATA-1-like domain [30]. At the same time, the FRET-efficiency was not high for the ATRX–MaSat pair, indicating that the association of ATRX with pericentromeric sequences may be mediated by other interactants. The spectrum of interactants mediating the association of ATRX with chromatin is rather extensive. However, based on the literary data, the most likely mediators in these interactions are HP1 and H3K9me3 [30,33].

Our data suggest that nuclear actin may also be involved in the recruitment of ATRX to heterochromatin in early mouse embryos. To date, it has been proven that actin is involved in a wide range of nuclear processes, including transcription and chromatin remodeling [34,35,36,37]. It has been biochemically established that nuclear actin is a component of chromatin remodeling complexes and also binds RNA polymerases and RNP complexes at transcription sites [35]. In mouse embryonic fibroblasts, the knockout of β-actin results in major chromatin rearrangements and the changes in histone modifications such as H3K9me3 [38]. It has been repeatedly reported that actin can interact with components of chromatin-remodeling complexes, modulating the activity of these complexes as a whole. For example, actin is present in chromatin-remodeling complexes BAF [39,40], INO80 [41,42], and SWR1 [43]. It is assumed that actin can regulate the function of the ATPase subunit in chromatin remodeling complexes and contributes to the recognition of extranucleosomal linker DNAs, forming a subcomplex with actin-related proteins [35].

We have previously shown that in regions of heterochromatin in two-cell mouse embryos, actin is present predominantly in oligomeric form [44]. Moreover, the intensity of actin labeling in the NPB-associated heterochromatin decreases significantly after DNase treatment, suggesting a direct interaction of this actin with DNA [45].

In morula nuclei, the association of ATRX with pericentromeric heterochromatin is reduced. This may be due to the first determination of cell fate, resulting in the trophectoderm (TE) and inner cell mass (ICM) cell lineages. The first stages of TE and ICM differentiation in mice are known to occur on the third day of development—during the compaction of the morula—when morula cells are divided into outer and inner cells that differ in morphology and polarity. The unpolarized inner cells express the factors of pluripotency, generating ICM cells in the blastocyst [46]. Already at the morula stage and possibly even earlier, core transcription factors associated with pluripotency—such as SOX2, OCT4, and NANOG—are activated in the ICM, while a homeobox/ParaHox transcription factor CDX2, which also plays a significant role in development, is activated in TE cells [47].

The dynamics of the ATRX distribution described above are typical for embryos developing in vivo—i.e., in natural, more favorable conditions. Under suboptimal in vitro culture conditions, this pattern changed significantly and differed markedly between embryos of the same biological age developing in enriched and simple culture medium. To analyze a possible relationship between embryonic viability and intranuclear distribution of ATRX, we used a model of the two-cell block in vitro [23]. Extensive research in the field of assisted reproductive technologies has led to the development of new complex culture media that allow the full preimplantation development of embryos [48,49]. After that, the in vitro developmental arrest began to be considered as an artifact due to the cultivation of embryos under suboptimal conditions. At the same time, we believe that this model provides ample opportunities for studying the mechanisms of early development, since blocked embryos retain their viability for a certain time. In the nuclei of arrested embryos, we found a significant change in the distribution pattern of ATRX and a decrease in the association of the protein with pericentromeric sequences.

An analysis of the molecular mechanisms underlying the change in the ATRX pattern in arrested embryos, as well as an analysis of the functional effects of ATRX redistribution, was not within the scope of our study. However, it seems likely that the main factor in changing the pattern of ATRX distribution in arrested embryos is the disruption of ZGA processes [18], which are known to include two successive waves of the enhancement of transcriptional activity. The major ZGA events in mouse development occur at the end of the second cell cycle [27], and the two-cell block in vitro is associated with pronounced changes in the normal pattern of transcriptional activity [50,51].

A change in the pattern of gene activity during ZGA is accompanied by morphological rearrangements of the embryonic nucleus associated with changes in the topology of certain genes. It should be emphasized that a significant number of genes important for embryonic development and activated during ZGA—e.g., *Zscan4* (Zinc finger SCAN domain containing 4)—are localized near the pericentromeric regions. Accordingly, the active or repressed status of such genes can affect the organization of these and nearby chromatin regions, which was confirmed using a modified method of FISH during the one–four-cell stage of mouse embryonic cleavage [52].

A delay of ZGA in embryos that are developmentally arrested at the two-cell stage [51] may affect the expression of at least some ATRX interactants, most of which are involved in maintaining chromatin structure (Supplementary Figure S2; Supplementary Tables S1 and S2). The change in the ATRX distribution pattern we described may thus be one of the manifestations of global rearrangements of the epigenetic landscape in the nuclei of blocked embryos caused by the disturbance of ZGA.

In conclusion, a comparative analysis performed for normally developing mouse embryos and embryos arrested at the two-cell stage showed that a decrease in the viability of mouse embryos and the probability of their successful preimplantation development is correlated with changes in the intranuclear distribution of ATRX. We currently cannot determine the character of this relationship, or whether it is direct or mediated by a change in the expression and/or functional activity of ATRX interactants. Nevertheless, we believe that the localization pattern of ATRX in the nuclei of early embryos can be considered as a criterion for their quality. Although this criterion cannot be used in clinical practice, such an assessment can be useful in experimental studies—e.g., when developing new media or protocols for working with oocytes and embryos.

## 5. Limitations of the Study

In the present study, a comparative analysis of the distribution of ATRX in the nuclei of mouse embryos developing in vivo and in vitro is limited to the use of morphological methods, mainly immunofluorescent microscopy, including FRET. This approach allowed us to analyze distribution patterns in individual embryos. However, it does not allow any conclusions to be drawn about the molecular mechanisms underlying changes in the normal distribution pattern of ATRX. Obviously, a solution to this problem using molecular genetic approaches is required in the future.

## Data Availability

The data presented in this study are available on request from the corresponding author.

## Supplementary Materials

The following supporting information can be downloaded at: https://www.mdpi.com/article/10.3390/life14010005/s1, Figure S1: Diagram illustrating experimental design; Figure S2: Functional ATRX protein–protein interaction network predicted by the STRING database; Table S1: ATRX functional partners predicted by String database; Table S2: GO enrichment analysis (biological processes) for predicted functional partners of ATRX.

## Appendix A

In this appendix, we present the results of additional immuno-FISH experiments demonstrating that ATRX is associated specifically with pericentromeric heterochromatin enriched in MaSat. When immuno-FISH was performed for ATRX and minor satellite DNA (MiSat)—a marker of the centromeric chromatin regions in mice [25,26]—the corresponding fluorescence signals were colocalized, suggesting that ATRX is not localized to centromeric heterochromatin (Figure A1).

Figure A2 shows the results of the simultaneous detection of ATRX, DAXX and MaSat in a two-cell mouse embryo. Although DAXX is a major partner of ATRX, including in early development [9], there is a high degree of colocalization between ATRX and MaSat, but not DAXX and MaSat.
