# Supplementary material for: Nuclear Distribution of the Chromatin-Remodeling Protein ATRX in Mouse Early Embryos during Normal Development and Developmental Arrest In Vitro
Source: Life (Basel). 2023 Dec 19;14(1):5. doi: 10.3390/life14010005 (PMC10817635; doi:10.3390/life14010005)
Supplement: Supplementary file 1 [file life-14-00005-s001.zip › Figure S2.pdf]

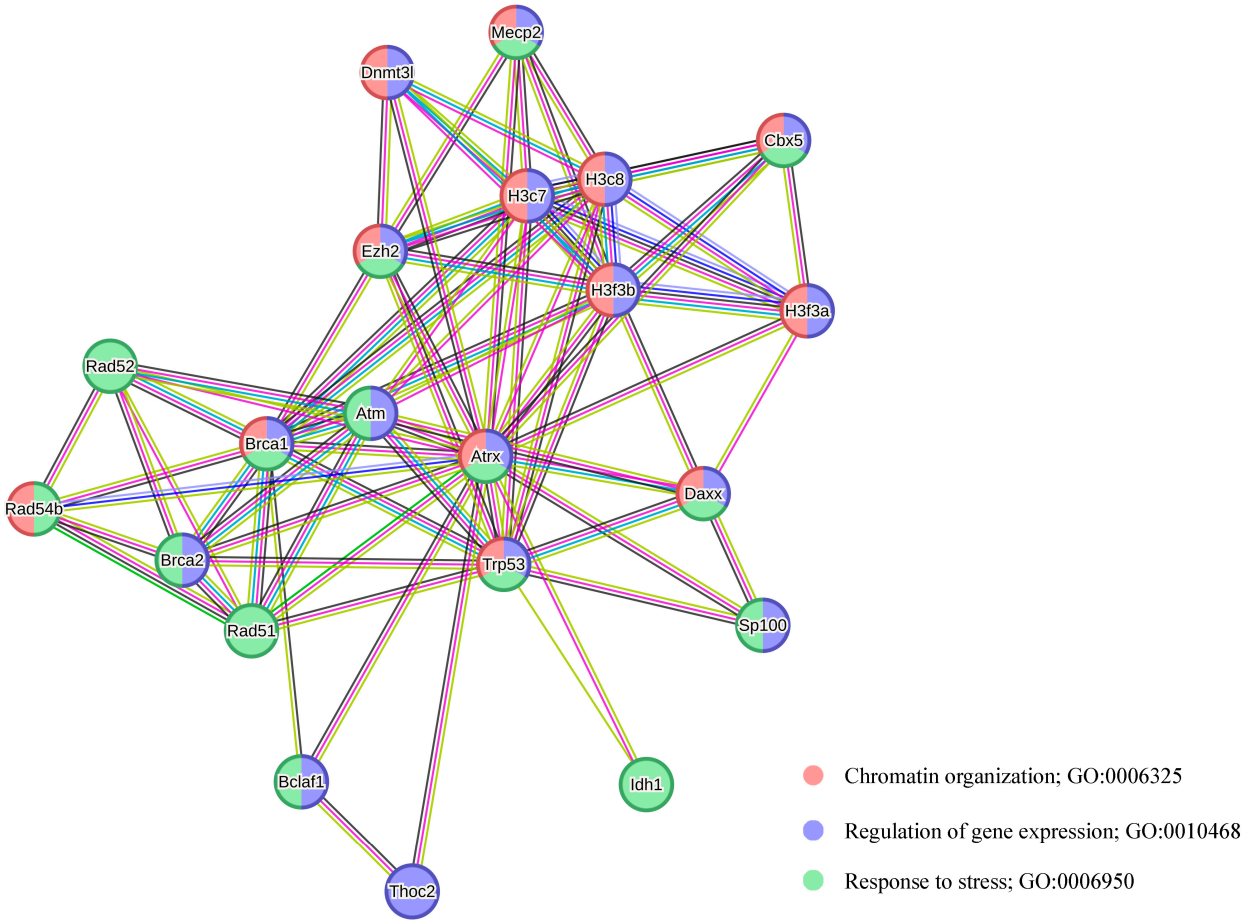

Figure S2. Functional ATRX protein-protein interaction network predicted by the STRING database (https://string-db.org/).
