# Supplementary figures and images for: Nuclear Distribution of the Chromatin-Remodeling Protein ATRX in Mouse Early Embryos during Normal Development and Developmental Arrest In Vitro
Source: Life (Basel). 2023 Dec 19;14(1):5. doi: 10.3390/life14010005 (PMC10817635; doi:10.3390/life14010005)

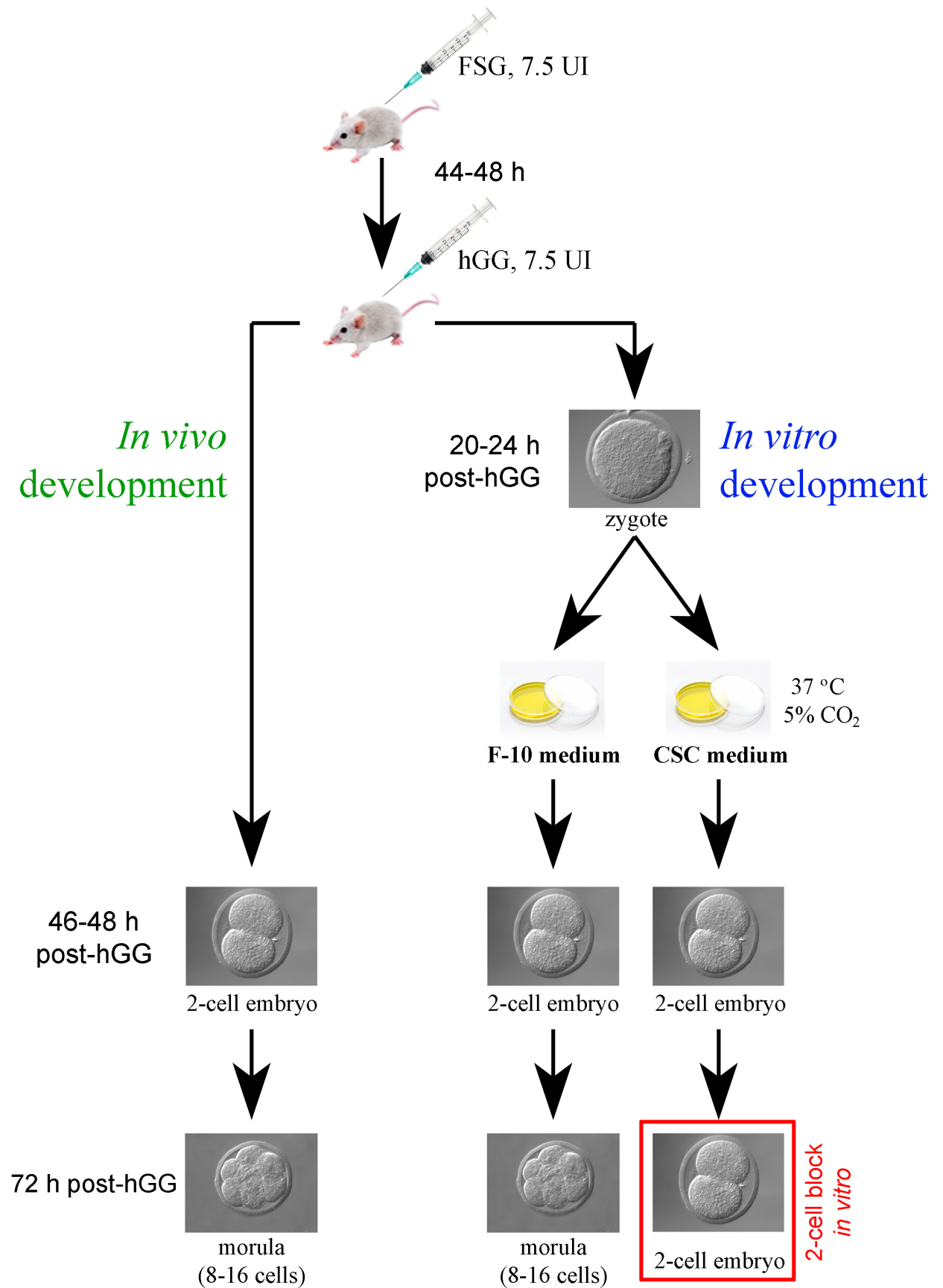

**Figure S1.** Diagram illustrating experimental design.

Supplement: Supplementary file 1 [file life-14-00005-s001.zip › Figure S1.pdf]
